# Rapid Quantification Method for Yield, Calorimetric Energy and Chlorophyll *a* Fluorescence Parameters in *Nicotiana tabacum* L. Using Vis-NIR-SWIR Hyperspectroscopy

**DOI:** 10.3390/plants11182406

**Published:** 2022-09-15

**Authors:** Renan Falcioni, Thaise Moriwaki, Werner Camargos Antunes, Marcos Rafael Nanni

**Affiliations:** Programa de Pós-Graduação em Agronomia, Department of Agronomy, State University of Maringá, Av. Colombo, 5790, Maringá 87020-900, Brazil

**Keywords:** hyperspectral reflectance, photochemical analysis, PLSR analysis, predictive model

## Abstract

High-throughput and large-scale data are part of a new era of plant remote sensing science. Quantification of the yield, energetic content, and chlorophyll *a* fluorescence (ChlF) remains laborious and is of great interest to physiologists and photobiologists. We propose a new method that is efficient and applicable for estimating photosynthetic performance and photosystem status using remote sensing hyperspectroscopy with visible, near-infrared and shortwave spectroscopy (Vis-NIR-SWIR) based on rapid multivariate partial least squares regression (PLSR) as a tool to estimate biomass production, calorimetric energy content and chlorophyll *a* fluorescence parameters. The results showed the presence of typical inflections associated with chemical and structural components present in plants, enabling us to obtain PLSR models with R^2^_P_ and RPD_P_ values greater than >0.82 and 3.33, respectively. The most important wavelengths were well distributed into 400 (violet), 440 (blue), 550 (green), 670 (red), 700–750 (red edge), 1330 (NIR), 1450 (SWIR), 1940 (SWIR) and 2200 (SWIR) nm operating ranges of the spectrum. Thus, we report a methodology to simultaneously determine fifteen attributes (i.e., yield (biomass), ΔH°area, ΔH°mass, Fv/Fm, Fv’/Fm’, ETR, NPQ, qP, qN, ΦPSII, P, D, SFI, PI_(abs)_, D.F.) with high accuracy and precision and with excellent predictive capacity for most of them. These results are promising for plant physiology studies and will provide a better understanding of photosystem dynamics in tobacco plants when a large number of samples must be evaluated within a short period and with remote acquisition data.

## 1. Introduction

Plants perceive light in the surrounding environment in very specific and sensitive ways, which induces biochemical, physiological and morphological changes at the individual level [1,2]. Light perception is considered one of the major environmental cues. This is important because it controls photosynthesis and adjustments at the electron transport chain in chloroplasts, influencing development and many mechanisms of growth and carbon fixation machinery. In addition, it regulates development to better accumulate carbon and energy in unstable and at distinct environments [3,4,5,6].

Photosynthetic regulation is highly sensitive to PSII activity. Additionally, the dynamics of light in the environment allow the plant to respond to abiotic and biotic stressors. This monitoring is a relevant technique not only for understanding photosynthetic regulatory mechanisms or dynamics of photosystems but also as a promising indicator of how plants respond to light environmental changes [7,8,9,10,11]. Among many available tools, chlorophyll *a* fluorometer devices, such as pulse-amplitude-modulation (PAM) fluorometry in conjunction with the saturation pulse method, remain the most utilized approaches [7,12,13,14]. Those methods consist of acclimating a leaf to the dark until all the reaction centers are “open” (i.e., oxidized) and then applying the leaf to a rapid and highly saturating light pulse. This approach induces to a progressive “closure” (i.e., reduction) of PSII reaction centers, resulting in an increase in the yield of chlorophyll *a* fluorescence to the electron transport chain [9,11,15,16,17]. In this way, the fluorescence of the chlorophyll *a* level starts to develop with a decrease again through a phenomenon termed “fluorescence quenching” that has two explanations according to [18]: (I) an increase in the rate by which electrons are transported away from PSII due to the light-induced activation of enzymes involved in carbon metabolism (Calvin–Benson cycle), such as photochemical quenching, and (II) an increase in the efficiency by which energy is converted to heat or thermal phenomena (i.e., “non-photochemical quenching”) [7,9,18,19,20,21].

Chlorophyll *a* fluorescence (ChlF) is a phenomena of the photosynthesis apparatus to cope with excess light energy accompanied by photochemical reactions, and heat dissipation or energy is funneled to the electron transport chain [7,18,22]. When the maximum photosynthetic rate is impaired, ChlF increases in many non-optimal environments [9,11,23,24]. Therefore, ChlF is a direct indicator of electron transport and photosynthetic activity and the most important tool for monitoring the status and dynamics of photosystems [7,15,21,25]. ChlF parameters are widely used to express the energy transfer to plant photosynthesis. The ChlF variables were calculated using standard (and largely discussed and acceptable) methodologies [9,20,21,22,24,26,27].

In contrast to the traditional approach, remote sensing analysis of chlorophyll *a* fluorescence rapidly detects the specific and immediate changes in a plant’s physiological status and response to various qualities of light treatments, heat, water conditions, photosystem reactions, and other biochemical and physiological processes [11,18,28]. Among all the ChlF parameters, Fv/Fm, which is the ratio of variable to maximal fluorescence, is related to the initial maximal efficiency of photons captured by open photosystem II (PSII) reaction centers in thylakoids and is a widely used parameter representing the dynamics of the health, growth and status of light reactions [9,11,18]. In light reactions, the Fv’/Fm’ parameter measures the efficiency of energy harvesting by partially closed (operating) PSII reaction centers exposed to light. It is an indicator of physiological plant stress, nutrients, and health status, as well as the dynamics of the energetic contents and status of molecules and structures present in the chloroplast [11]. Both Fv/Fm and Fv’/Fm’ are closely related to the current activity of plant photosynthetic performance and have been applied to monitor photosynthesis and physiological performance and their metabolic reactions depending on the light in the environment [18,29,30,31,32].

A hyperspectral sensor based on spectroscopy measurements is a high-throughput sensor technology that can be used to monitor the optical properties of living vegetation (e.g., leaf and canopy reflectance) and enables the rapid and non-destructive assessment of plant status, along with a simultaneous estimate of several plant traits in the field for a large number of plants over multiple periods [3,7,22,33,34,35]. The prediction of these parameters from leaf spectra is based on the vibrational relationships of light with molecular organic bonds, mainly -C-H, -N-H, -COOH, -NH_3_ and -O-H. These result in vibrational excitation at specific wavelengths through the visible (Vis: 400–700 nm), near-infrared (NIR: 700–1100 nm) and shortwave infrared (SWIR: 1100–2400 nm) spectral regions [3,4,7,22,36].

An additional ever-expanding approach is the use of multivariate statistical methods to directly model commonly used plant attributes as a function of the hyperspectral reflectance profiles [7,28,37]. Advances in the sensitivity and portability of hyperspectral radiometer devices, as well as in computational capacity and multivariate tools for modeling (e.g., using partial least squares regression, PLSR, STEPWISE, principal component analysis (PCA), and other statistical methods) [3,4,28], allow some advances in monitoring the plant status. Thus, the use of this approach to estimate a variety of commonly investigated plant parameters and physiological processes based on leaf optical properties, including morphological, physiological and biochemical parameters, is currently possible [26,34,38,39]. For this purpose, a calibration model is developed by pairing leaf spectra collected based on an uniform, calibrated and direct light source in a consistent manner with independent and reliable reference methods for measuring samples. Subsequently, the model development is validated by comparing relationships between observed and predicted values collected from other independent samples, databases or environments of experiments [4,7,11,36]. This calibration model is then used to predict the variable of interest in unknown samples based on their spectral signature. Currently, hyperspectral analysis uses the full spectrum for the analysis of many parameters in plants [7,22,33,36,40] instead of “key” wavelengths for vegetation indices.

Considering the needs described above, the aim of this study was to evaluate the capacity to predict attribute yield, calorimetric energy and chlorophyll *a* fluorescence parameters in tobacco plants as a model system using hyperspectral reflectance curves in the Vis-NIR-SWIR range. For this, full reflectance spectra of tobacco plants were obtained using a Vis-NIR-SWIR spectroradiometer (400–2400 nm) as a rapid dataset to estimate 15 principal attributes to monitor plant status, namely, yield (biomass), ΔH°area, ΔH°mass, Fv/Fm, Fv’/Fm’, ETR, NPQ, qP, qN, ΦPSII, P, D, SFI, PI_(abs)_, D.F. If successful, hyperspectral reflectance spectroscopy should be used directly to estimate several widely used chlorophyll *a* fluorescence parameters in tobacco plants with higher accuracy and precision of predicted models.

## 2. Results

### 2.1. Descriptive Analysis

The descriptive analysis of the yield, calorimetry energy and chlorophyll *a* fluorescence parameters of four (4) light qualities (white, blue, red and red added to far-red) for tobacco growth is shown in Table 1. Coefficients of variation (CVs) ranged from 3.7 to 65.04% (Table 1). In addition, [41] suggests the following CV classification: low (<10%), medium (between 10 and 20%), high (between 20 and 30%) and very high (>30%). Thus, 11 of the 15 parameters analyzed here show CVs (%) classified as medium to very high, with five medium parameters (ETR, qP, qN, ΦPSII, P and D.F.), one high parameter (SFI) and five very high parameters (yield, ΔH°mass, ΔH°area, NPQ and PI_(abs)_) (following abbreviation list).

### 2.2. Hyperspectral Reflectance Analysis

**Figure 1:**
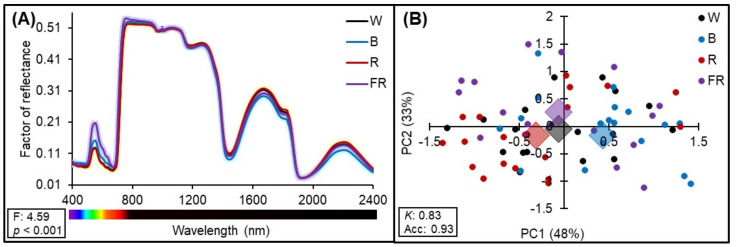
(**A**) Average factor of reflectance profile of tobacco leaves grown at different light qualities. White (W), blue (B), red (R) and red added to far-red (FR); F and P values from permutation analysis of variance (PERMANOVA) for the effects of light qualities on the full range (400–2400 nm) of hyperspectral reflectance to tobacco leaves are reported in the bottom-left corner of the panel. (**B**) Principal component analysis score for the first (PC1) and second (PC2) principal components of the hyperspectral reflectance data (400–2400 nm) collected from tobacco plants. Average accuracy (Acc) and *Kappa* (K) values from partial least squares regression (PLSR) are reported in the bottom-left corner of the panel.

Raw leaf hyperspectral reflectance data for the four light qualities used for cultivation (260 samples; average of the reflectance data) are shown in Figure 1. PERMANOVA analysis was used to discriminate significant wavelengths (F: 4.59; *p* < 0.001) (Figure 1A) from the spectra. A slight (and significant) variation in reflectance factor intensity was observed, especially in the visible (Vis) region (400–700 nm), due to leaf pigments such as chlorophyll and carotenoids, and in the near-infrared (NIR) region (700–1100 nm), due to structural differences in the leaf mesophyll. The majority of functional groups (molecular vibrational) detected in the shortwave infrared (SWIR) region (1100–2500 nm) showed differences between treatments (Figure 1A). The higher classification, which was based on the PCA score (PC1: 48% and PC2: 33%) of light qualities from the spectra (i.e., high mean *Kappa* (K)) identified by PLSR, was observed with calibration:cross-validation and 70:30 ratio for calibration:validation data using the 15 components, with a *Kappa* value (K) of 0.83 and accuracy (Acc) of 0.93 (Figure 1B).

Despite the apparent small difference in the hyperspectral reflectance range (400–2400 nm) between treatments, no misclassification occurred between the light-grown tobacco plants (Figure 1).

### 2.3. Principal Component Analysis (PCA)

**Figure 2:**
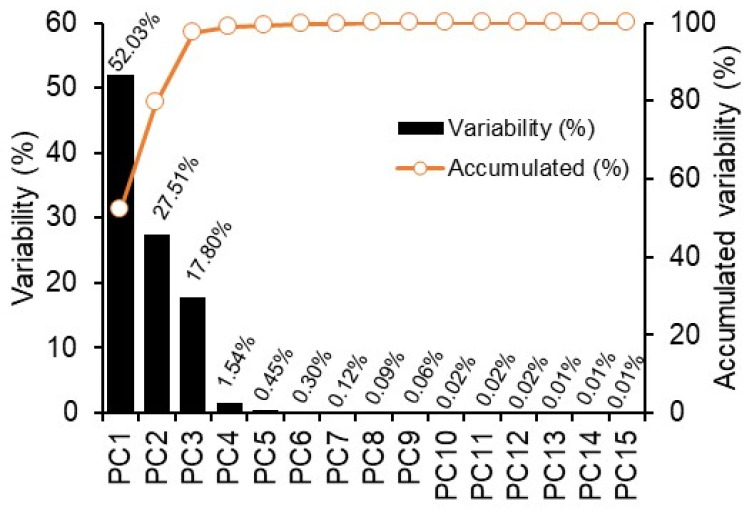
Principal component analysis (PCA) and PC scores obtained from hyperspectral analysis of the tobacco plants. Black bars indicate variability of PC, and orange circles indicate accumulated variability.

Fifteen (15) main components were obtained from the spectrum using PCA (Figure 2). The first and second principal components (PC) represent 79.5% of the total variance that can be explained. For instance, some analyses performed as a factor of PLS may use different numbers of PCs ranging from 2 for NPQ to 11 for ΔH°. However, these 15 PCs, were simulated by testing maximum combinations as much as possible.

### 2.4. Prediction of Yield, Energy Calorific and ChlF Parameters

Statistical metrics of the PLSR models from the calibration (Cal) and cross-validation (Cva) methods for yield, energy calorific and ChlF parameters are shown in Table 2. Regarding hyperspectral data collected from 400–2400 nm, a distinct difference in behavior was observed between the phases (Cal and Cva). For the eight best results of R^2^_CV_ and RPD_CV_, yield, ΔH°mass, ΔH°area, Fv/Fm, ETR, qP, ΦPSII and P displayed very good and excellent prediction scores, respectively. The best results for R^2^_CV_ and RPD_CV_ metrics were obtained using full hyperspectral data (400–2400 nm) (R^2^_CV_ > 0.82, RPD_CV_ > 2.0). Regarding bias, similar results (close to zero) were obtained for all techniques evaluated (data not shown).

The prediction models for tobacco parameters were adjusted according to the number of PLSR factors previously tested by cross-validation. Thus, the relationship between predictor (reflectance) and predicted (tobacco parameters and ChlF) variables was better explained using models containing 5 factors for yield; 7 factors for Fv/Fm and qP; 8 factors for ETR, ΦPSII, and P; and 10-11 factors for ΔH°area and ΔH°mass (Table 3).

**Figure 3:**
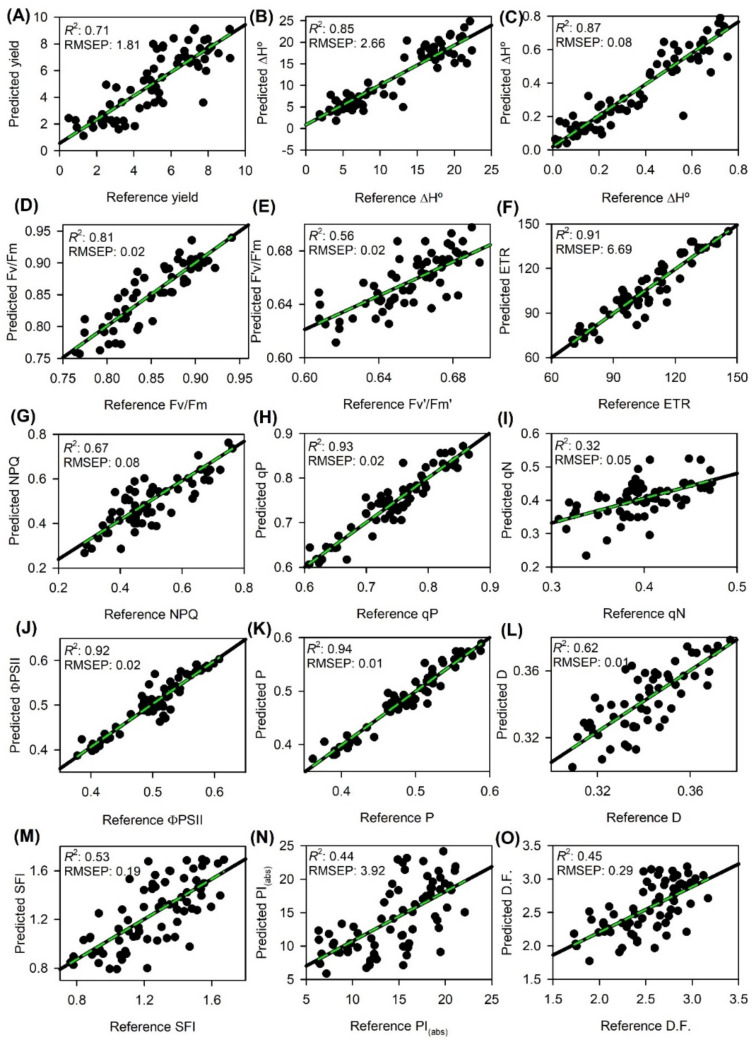
Reference vs. predicted data estimated by partial least squares regression (PLSR). Dotted (green) and solid (black) lines represent regression and 1:1, respectively. Model goodness-fit (R*^2^*) and root mean square error predicted (RMSEP) for validation data generated using 70% of the data for calibration and 30% for validation are reported. Bias outputs are not shown, as they were always lower than 0.01. (**A**) Yield, biomass of tobacco plants (g plant^−1^); (**B**) ΔH°mass (kcal g^−1^), calorimeter energy content by mass; (**C**) ΔH°area (kcal m^−2^), calorimeter energy content by area; (**D**) Fv/Fm: Maximum quantum efficiency of PSII photochemistry in the dark-acclimated state; (**E**) Fv’/Fm’: Maximum efficiency of PSII in light-acclimated state; (**F**) ETR, electron transport rate (µmol m^−2^ s^−1^); (**G**) NPQ, non-photochemical quenching calculated as (Fm − Fm’)/Fm’; (**H**) qP: Photochemical quenching calculated as (Fm’–Fs)/(Fm’ − Fo’); (**I**) qN: Non-photochemical quenching calculated as (Fm − Fm’)/(Fm − Fo’); (**J**) ΦPSII, PSII operating efficiency in light conditions as calculated (1 − Fs/Fm’); (**K**) P, the fraction of light absorbed in PSII antennae that is utilized in PSII photochemistry; (**L**) D, fraction of light absorbed in PSII antennae that is dissipated thermally; (**M**) SFI, Structure function index; (**N**) PI_(abs)_, Performance index for energy conservation from photons absorbed by PSII antenna, until the reduction of PSI acceptors; (**O**) D.F., Driving force on absorption light by cross-section leaf.

The PLSR models, according to the R^2^_CV_, were considered very good (0.81–0.88) for yield and ETR and excellent (0.91–0.93) for ΔH°mass, ΔH°area, ΦPSII, qP and P. According to the RPD_P_ metric, the models were considered excellent (>2.29) for all variables evaluated (Table 3; Figure 3).

Regarding the RMSE_CV_ of each variable, this metric was close to RMSE_P_ (RMSE_CV_ ≈ RMSE_P_); in some cases, it was slightly smaller, while it was slightly higher in others (Table 2 and Table 3; Figure 3). The values of bias tended to be zero for all parameters in the cross-validation and prediction analyses (data not shown).

All 15 proposed parameters were adjusted with an independent dataset (hyperspectral data) from those used in the cross-validation phase to assess the ability of PLSR models to predict these parameters. The scatter plots of the predicted versus reference data, including the results of the multivariate statistics metrics, are shown in Figure 3.

The prediction models for tobacco parameters were adjusted according to the number of PLSR factors previously tested by cross-validation. Thus, the relationship between predictor (reflectance) and predicted (tobacco parameters and ChlF) variables was better explained using models containing 5 factors for yield; 7 factors for Fv/Fm and qP; 8 factors for ETR, ΦPSII, and P; and 10–11 factors for ΔH°area and ΔH°mass (Table 3).

### 2.5. Regression Coefficients (RCs) and Variable Importance in Projection (VIP)

**Figure 4:**
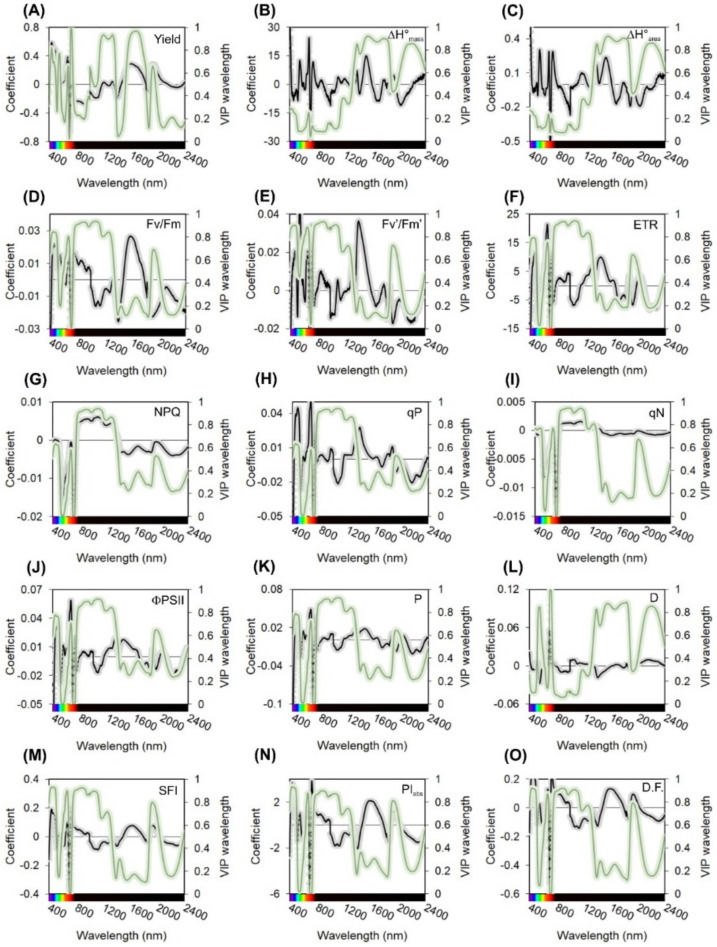
Mean and standard error of regression coefficients (RCs) and variable importance to the projection (VIP) methods. (**A**) Yield, biomass of tobacco plants (g plant^−1^); (**B**) ΔH°mass (kcal g^−1^), calorimeter energy content by mass; (**C**) ΔH°area (kcal m^−2^), calorimeter energy content by area; (**D**) Fv/Fm: Maximum quantum efficiency of PSII photochemistry in the dark-acclimated state; (**E**) Fv’/Fm’: Maximum efficiency of PSII in light-acclimated state; (**F**) ETR, electron transport rate (µmol m^−2^ s^−1^); (**G**) NPQ, non-photochemical quenching calculated as (Fm − Fm’)/Fm’; (**H**) qP: Photochemical quenching calculated as (Fm’ − Fs)/(Fm’ − Fo’); (**I**) qN: Non-photochemical quenching calculated as (Fm − Fm’)/(Fm − Fo’); (**J**) ΦPSII, PSII operating efficiency in light conditions as calculated (1 − Fs/Fm’); (**K**) P, the fraction of light absorbed in PSII antennae that is utilized in PSII photochemistry; (**L**) D, fraction of light absorbed in PSII antennae that is dissipated thermally; (**M**) SFI, Structure function index; (**N**) PI_(abs)_, Performance index for energy conservation from photons absorbed by PSII antenna, until the reduction of PSI acceptors; (**O**) D.F., Driving force on absorption light by cross-section leaf.

The regression coefficients (RCs) and variable importance in projection (VIP) metrics of the PLSR model are shown in Figure 4. Regions of peaks and valleys where the RC and VIP exerted a substantial effect on the construction of the prediction model were generally well distributed among all spectra (Vis-NIR-SWIR).

The RC and VIP values used for PLSR models vary between 5 and 17 wavelengths (peak and valley) resulting from higher RCs in regions close to 400 (violet), 440 (blue), 550 (green), 670 (red), 700–750 (red edge), 1330 (NIR), 1450 (SWIR), 1940 (SWIR) and 2200 (SWIR) nm (Table 4; Figure 4). Although the NPQ and qN parameters used five VIP values, NPQ had high prediction values, while qN did not show a similar performance (Table 3 and Table 4; Figure 3). In general, parameters that obtained RPDs higher than 3.33 (Table 3) (ETR, qP, ΦPSII, and P) obtained excellent predictions for VIP-selected wavelengths (Table 4).

## 3. Discussion

### 3.1. Descriptive Analysis

**Figure 5:**
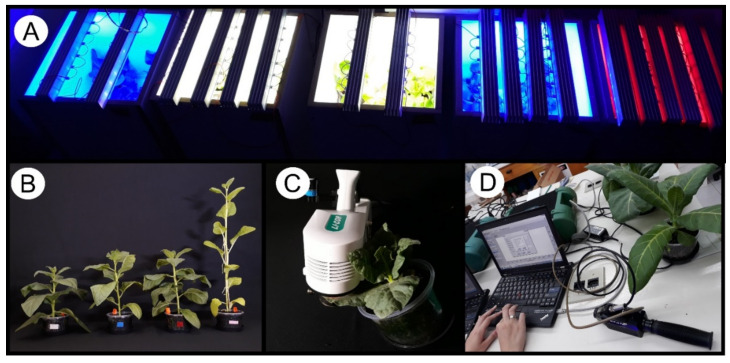
Experimental procedures. (**A**) Box used to grow plants with controlled light quality and intensity. White LED light (W), blue (B), red (R) and red added to far-red (FR); (**B**) random samples of tobacco plants at 35 days old used for sampling. (**C**) “Standard” measuring chlorophyll *a* fluorescence parameters by infrared gas analyzers (IRGAs; LI-6800, Li-Cor Inc., Lincoln, NE, USA). (**D**) Measuring hyperspectral reflectance by ASD Fieldspec^®^ (ASD Inc., Boulder, CO, USA).

**Figure 6:**
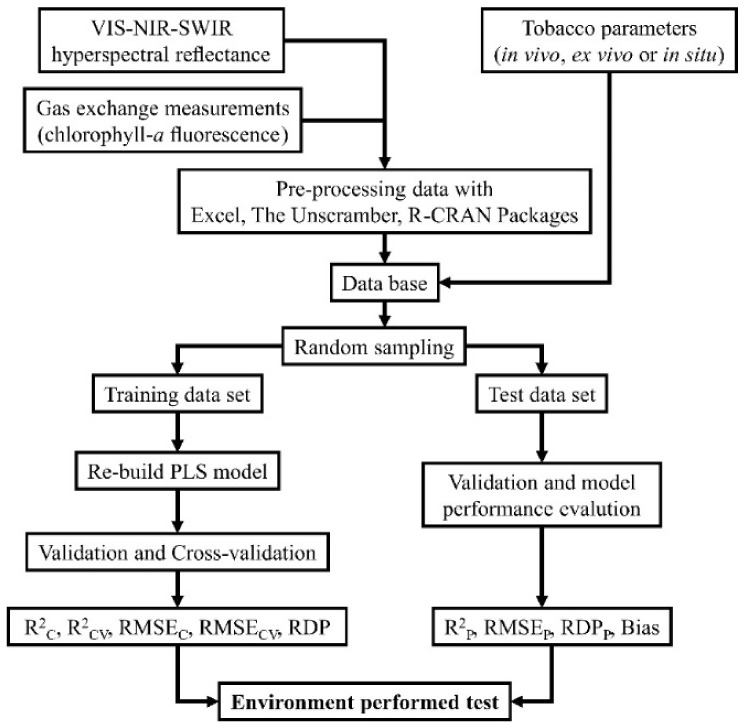
Flowchart of the methodology used in the PLSR analysis. Abbreviations—see Table 1 or abbreviation list.

The variability observed in tobacco plants grown under light with different qualities (Figure 5) enables an efficient estimation of parameters such as biomass (yield), energetic contents (ΔH°area and ΔH°mass), and ChlF (Fv/Fm, Fv’/Fm’, ETR, NPQ, qP, ΦPSII, P, and D) based on the 400–2400 nm spectrum (Figure 4). Some parameters, such as qN, SFI, PI_(abs)_ and D.F., were classified as having low and moderate prediction accuracy (low RPD values) (Table 3). All approaches used here (Figure 6) enable a rapid assessment with high-throughput measurements that produce data-rich results. In particular, remote sensing fluorescence techniques should play a key role in the development of simple, fast and efficient prediction crop phenotyping in response to photodevelopment and monitoring the dynamic status of photosynthesis and photosystem dynamics under different environmental conditions [7,9,28,35]. In general, after comparing the W, B, R, FR groups, the photobiology was associated with high plastic development in response to light (Figure 5). In addition, the collection of data under different physiological conditions with distinct photochemical efficiencies and chlorophyll *a* fluorescence quenching enabled large differences between minimum and maximum values to be observed.

### 3.2. Analysis of the Reflectance Spectrum

The differences among the Vis to NIR to SWIR bands were detected in the all-hyperspectral curve. Vis exhibits inflections arising from the absorption of pigments, such as chlorophylls and carotenoids, which are more intensely related to the levels of these pigments. In addition, many compounds and proteins of photosystems (i.e., plastocyanin, plastohydrochinone, ATP-synthases, and oxygen-evolving complex) contribute to the absorbance of blue, green and red bands (Vis), as well as NIR and SWIR light [42,43,44].

In the NIR region, the higher reflectance values and the sharp differences in anatomical and physiological traits of plants [45,46] between light with distinct qualities are related to radiation scattering within chloroplast and mesophyll cells [4,34,36]. Thus, during stress, the plant begins to exhibit reduced vigor, the mesophyll often deforms, photosystem efficiency is reduced, and the presence of misfolded proteins causes reflectance to be reduced [47,48]. In particular, tobacco plants, which are quite plastic regarding the structure and thickness of their mesophyll, as well as the biochemical properties, compounds and accumulated calorific energy of their leaves, have different reflectance spectra, mainly in the NIR region, which are strongly influenced by these characteristics. From this perspective, tobacco plants contain a significant amount of pigments (carotenoids, chlorophyll, phenolic compounds), polyphenols and fatty acids common in triglycerides, oleate and linoleate or other molecules and metabolites in the vacuole, cytoplasm and plasma membrane that can be quantified by sensors operating in the NIR region. Thus, NIR was an important band for quantification and monitoring of the status of dynamics of electron transport chains (ETR, qP, NPQ, Fv/Fm and Fv’/Fm’) induced by photochemical and non-photochemical quenching and specific parameters, as highlighted in the most responsive VIP values shown in Table 4.

In the SWIR region, significant differences were observed in the spectral analysis. Hyperspectral data in this region contribute to obtaining fingerprints, especially at 1450 and 1900 nm, which are important for the characterization of water bonds and other compounds that contain hydroxyl groups, such as sugars, hemicellulose, pectin, cellulose and lignin [35,49,50]. Many of these compounds are associated with a higher energetic status (enthalpy variation in vibration or energy of molecules), the demand for biosynthesis and cost construction. Wavelengths of 1120, 2130 and 2300 nm typically promote -C-H and -NH stretching from aromatic rings, which are associated with the main compounds contained in tobacco, namely, sugars, alkaloids, and proteins associated with high calorific energy (ΔH°).

When all hyperspectral curves were applied to distinguish a particular variable, the effects of several background errors on misclassification were reduced, in relationwhen only some wavelength bands were used. Thus, when applied, the all/full spectrum may improve the reliability and accuracy of the analysis to better generate a prediction model. Similarly, [51] reports that the effects of various background interference sources can be reduced or eliminated when using the entire curve compared to using only a few peaks [45,52]. Since the degree of scattering depends on the wavelength, refractive and/or reflexive index, full hyperspectral data compensate for additive and multiplicative effects on spectral data induced by nonuniform scattering. In general, the full spectrum, in contrast to a few range-specific bands, is the more appropriate method for Vis-NIR-SWIR analysis to predict parameters with higher accuracy and precision.

### 3.3. Partial Least Squares Regression (PLSR)

The results of R^2^_CV_, R^2^_P_, RPD_CV_, RPD_P_, RMSE_CV_ and RMSE_P_ for the full spectrum of bands (400–2400 nm) used varied according to the parameters evaluated (Table 2; Figure 3). In general, the approach used here (Figure 6) produced very good results. Other techniques and multivariate statistical analysis might achieve similar predictive results using STEPWISE, regression coefficients (RCs), variable importance to the projection (VIP) methods or processes with noise reduction efficiency. For example, multiplicative scatter correction (MSC), Savitzky–Golay (SG) and standard normal variate (SNV) methods vary according to the attribute measured but have been used alternatively to obtain good results, as reported elsewhere [5,13,24,53].

Inclusion of vibrational modes induced by SWIR, such as -C-H, -N-H, and -NH_3_ stretching vibrations from aromatic rings associated with specific wavelengths and related to compounds along with the structure of the mesophyll cell wall, proteins and fatty acids, allowed us to obtain better PLSR models with statistical metrics, such as R^2^ and RPD, ranging from good to excellent, respectively, for all evaluated attributes [4,35,42,54].

In the cross-validation phase, the results for the statistical metrics were slightly higher than those obtained in the prediction phase, as expected, since the number of samples used to obtain the model was smaller in the calibration phase [34]. In addition, [34,36] used NIR-SWIR spectroscopy to predict tobacco attributes and similarly obtained an increase in RMSE in the prediction phase.

In the prediction phase, the R^2^_P_ values obtained for the ChlF variables (Fv/Fm, Fv’/Fm’, ETR, NPQ, qP, qN, ΦPSII, P, D, SFI, PI_(abs)_ and D.F.), were similar to other variables from tobacco [34,42], *Phoenix dactylifera* [7], wheat [11] and *Suaeda salsa* [35] employing Vis and/or NIR and/or SWIR spectroscopy. In the present study, the difficulty of establishing metrics with better results for the yield, energetic calorimetry and ChlF variables, especially in the prediction phase, was possibly due to the limited number of samples (*n* = 78). The authors of a previous study [7] used specific range bands and were able to define R^2^_P_ with better metrics. However, when they set specific spectral peaks or valleys, the prediction models showed high precision but possibly lower accuracy [4,5,55]. Therefore, due to complexity and occurrence from dark to 1 s induction, we were unable to obtain more robust data capable of capturing all the variability from JIP-test attributes (SFI, PI_(abs)_ and D.F.; Table 3) [56].

In this research project, the high accuracy of the model to estimate attributes with Vis-NIR-SWIR spectroscopy proved to be useful (or excellent according to the RPD_P_) for most of the variables tested. Thus, the main advantage of the method we propose is the ability to easily predict simultaneous inferences from fifteen variables, namely, yield, ΔH°area, ΔH°mass, Fv/Fm, Fv’/Fm’, ETR, NPQ, qP, qN, ΦPSII, P, D, SFI, PI_(abs)_ and D.F. This information is important for monitoring/understanding the photosynthesis status in a single collection in a non-invasive manner (avoiding sample loss) without the need for preparation using reagents or expensive and high-cost equipment for acquisition [4,36].

### 3.4. Regression Coefficients

Deep interpretation of regression coefficients (RCs) and variable importance in projection (VIP) is essential to avoid possible erroneous correlations, as highlighted. Thus, we aimed to better understand how each variable (wavelength) contributed to the significant variation in the model used to estimate the parameters. In most cases, high RC (Figure 4) and VIP wavelengths (Table 4; Figure 4) were well distributed throughout the three portions of the spectrum (Vis-NIR-SWIR) for all attribute methods analyzed [4,7,34,36].

Some studies have investigated the potential of remote sensing as a technique for estimating tobacco biomass production, and ChlF attributes primarily considering the Vis-NIR or just the NIR region [34,35,53], where biased estimates were obtained for parameters or low-accuracy outputs. In the present study, the high regression coefficients in the SWIR region highlight the importance of this region for predicting attributes (on average, 30% of VIP values), as indicated in the hypothesis of this study. A previous study [22] states that differences in leaf structure and composition are associated with SWIR reflectance. According to [36], the use of reflectance in the SWIR region provides important information to define the photosynthetic potential of tobacco plants. In addition, the calorimetric energy (ΔH°) shows a stronger correlation with the energy content in these molecules and structures present in electron chain transport, cell structures, and other proteins and enzymes of oxidative systems of plants, with the importance of SWIR reaching 41% of VIP values [22,34,36].

Regarding the yield variable (Figure 4), the high RC and VIP values at 440 and 672 nm are related to the absorption of blue and red light by chlorophyll, respectively. The value obtained at 550 nm is associated with minimal absorption (valley) of green light by chlorophyll. We highlight that the absorption of 550 nm light was influenced by the chlorophyll content [57] and leaf thickness [2]. In addition, the chlorophyll content is related to biomass accumulation in plants [3,22,36]. High RC and VIP values close to 1120 nm are related to the cell wall as cellulose/lignin/hemicellulose/pectin [36,58,59]. The bands at 1940 and 2300 nm, in turn, are possibly due to the constituent proteins (N-H bend + C=O stretch/N-H in plane banding/C-N stretch combination bands) of plants [3,59,60], which may also be related to biomass production in tobacco leaves.

Regarding the photochemical efficiency parameters, such as Fv’/Fm’ and ETR, the higher RC and VIP values at approximately 400 and 670 nm are related to chlorophyll [2,28,61]. Those at 1440 nm are associated with typical features of asymmetric -N-H stretching vibrations, which are directly related to proteins [22,28]. These components (chlorophyll and proteins) are the basis of the light-harvesting complex in photosystems.

Concerning the calorimetric energy (ΔH°), as suggested by [62], Vis-NIR-SWIR energy allowed us to measure the chemical compositions of plant leaves non-destructively. We speculate that most environmental and metabolic factors influence the construction cost of plant tissues and contribute to alterations of ΔH°. In addition, when evaluating ΔH° (biomass and area) using Vis-NIR-SWIR spectroscopy, RC and VIP have been used to predict calorimetric energy using wavelengths in the blue, green, red and far-red bands of approximately 1100, 1500, 1900 and 2040 nm, respectively (similar to those used in this study and reported in other studies). In this sense, our results reaffirm the high accuracy and precision of the proposed models [3,52,62]. In addition, one of the goals of remote sensing is to be able to quantify data quickly, simply and efficiently, based on predictive models [63]. In this perspective, diversity metrics on spectral variance using mixed models and distinctive morphological and/or biochemical leaf traits [63,64] are highly necessary for a better understanding of the energetic dynamics and photochemical efficiency of plants [61].

Wavelengths in the NIR-SWIR regions are associated with the vibration modes of the first overtones of -C-H, -NH, -CH_3_ and -COOH functional groups. According to Shorten et al. (2019), wavelengths in the 900–1700 nm range provide a much better prediction of proteins than wavelengths in the 550–900 nm range, which was shown in this study [38]. In addition, the authors evaluated NIR-SWIR spectroscopy data to determine increased biomass (i.e., yield) and energy contents (ΔH°) in tobacco, ranging from 1002 to 1610 nm and 1815 to 2211 nm (similar to those found in our study). According to [39], the best wavelengths for defining total protein contents in rice, palm, Arabidopsis, poplar and other plants are 521, 524, 532, 553, 697, 718, 759, 1065 and 1993 nm [7,22,34,65].

Acquisition of full spectra using high-resolution sensors and analysis by curve deconvolution associated with PLSR [66] and other multivariate analyses [28,33] derive more robust and reliable models, as evidenced by the RMSE values that are related to the use of contiguous bands, to the detriment of the use of select specific bands that take advantage of specific wavelengths [2,4]. The flexibility of choosing between different spectral bands by performing a discriminant analysis associated with the high accuracy and precision of high-resolution spectral data enables species discrimination, pigment concentration estimations and, as reported here, predictions of many ChlF parameters, such Fv/Fm, Fv’/Fm’, ETR, NPQ, qP, ΦPSII, P and D [2,4,7,50,52].

### 3.5. Benefits and Limitations of Using Vis-NIR-SWIR Spectroscopy for Monitoring ChlF

The approach used in this research employing reflectance hyperspectroscopy allows us to accurately, rapidly and non-destructively screen the understanding of PSII efficiency to distinguish environmental constraints and other abiotic and biotic interactions. In addition, the ability to specifically distinguish among light-mediated regulation of development and photosystem dynamics is relevant to monitoring plant physiological states [4,7,22,36,58]. By combining hyperspectral reflectance data, standard ChlF measurements, and robust multivariate statistical modeling, this study described a new perspective method with the potential to concomitantly predict the spectral yield, calorimetric energy in leaves, and many ChlF parameters.

Many physiological research and leaf-to-canopy samples of the whole set of ChlF parameters are frequently challenging, because they currently require several minutes per leaf as the leaf must first reach the dark-adapted state (15–45 min at 4–12 h; depending on the plant genotype or species) and then return to the light-adapted state [4,7,11]. Spectral approaches have been shown to be a valid alternative to standard measurements of photosynthetic traits since they correlate with photosynthetic processes (e.g., the xanthophyll cycle, oxygen-evolving complex (OEC), electron transport chain between plastoquinone and plastocyanin transports, and energy dissipation measured as regulated and nonregulated photochemical and non-photochemical quenching) [11,13,35]. In addition, specific and common parameters of photosynthetic traits such as net photosynthesis, stomatal conductance (*g*_s_), the leaf maximum carboxylation rate (Vc_max_), and the maximum rate of electron transport (ETR) might be directly predicted from spectral data with high accuracy and precision [28,42]. Some attempts to evaluate the relations between spectral signatures and a few ChlF parameters have also been reported [7,34,36,53,62].

Thus, techniques involving remote sensing approaches (such as Vis-NIR-SWIR spectroscopy) are very useful and promising in regard to meeting this need, as they are fast and do not require prior sample preparation with chemical reagents or expensive equipment [4,36]. Some models created in this study were considered excellent for the parameters evaluated, suggesting that the use of the Vis-NIR-SWIR spectroradiometer is a promising strategy for understanding the dynamics of photosynthesis in tobacco plants.

Technical limitations were observed for JIP-test parameters such as SFI, PI_(abs)_ and D.F. However, these parameters, which presented low values for the prediction (R^2^_P_ = 0.45–0.53; RPD = 1.34–1.46), may be related to biological phenomena related to the transition from dark to light (often 1 s) that was not efficiently captured by reflectance measurements.

Considering the ease of collecting a single dataset sample (spectra) to simultaneously estimate complex attributes, such as estimated plant yield, energetic content and ChlF, the methodology proposed in this study was easy to implement. In addition, we expect other researchers to use this method to enhance our prediction capacity of lignin, cellulose, fatty acids, proteins specific to the photosystem complex, and other compounds that are also important for tobacco, as well as other crops [2,4,7,34,36].

## 4. Materials and Methods

### 4.1. Plant Material, Growth Conditions and Experimental Design

Experiments were conducted at the Plant Ecophysiology Laboratory, Department of Biology, at the State University of Maringá, Maringá, Paraná, Brazil. Tobacco (*Nicotiana tabacum* cv Samsun NN) plants were used. Seeds were germinated on Germitest^®^ paper immersed in 5 mL of Hoagland’s solution (pH 5.4) in a dish. After 30 days of growth, seedlings were transplanted from the commercial substrate and transferred to individual white-covered open-top wood boxes illuminated with low spectral dispersion LEDs (light emitting diodes) [white (W), blue (B), red (R) and red-plus-far-red (FR)], with light irradiance fixed at 200 µmol m^−2^ s^−1^, individually adjusted by a LI-190R quantum sensor (Li-Cor Inc., Lincoln, NE, USA) under a 12 h/12 h photoperiod (light/dark) at 25 °C (±3 °C) with 70% relative humidity. The experimental design was a random scheme with tobacco plants grown under an LED to produce a broad spectrum of datasets. On the 28th day after transplantation, the plants were collected and freshly analyzed, frozen in liquid N_2_, stored at −80 °C, and freeze-dried or oven-dried (70 °C) for the various analyses (Figure 5 and Figure 6).

LED emissivity was verified using a high-resolution spectroradiometer (ASD Inc.; FieldSpec 3, Boulder, CO, USA). White (W—peak at 443 and 580 nm), blue (B—peak at 450 nm), red (R—peak at 658 nm) and red added to far-red (FR—peak at 742 nm) values (data not shown) were measured as described in a previous study [58,59].

### 4.2. Growth and Yield Analysis

Destructive growth analyses were conducted on days 30–35 after seedling transplantation as described following [61,67]. Leaf-blade dry weight (DW) was obtained using a forced ventilation oven for 72 h at 70 °C. Leaf area (LA) was obtained using an LI-3100C leaf area meter (Li-Cor Inc., Lincoln, NE, USA).

### 4.3. Calorimetric Analysis

A calorimetric energetic analysis (ΔH°) was performed to evaluate the differential construction costs of leaf plant tissue. For the quantification of the calorimetric content, the dry matter of leaves was oven-dried at 70 °C until reaching a constant weight. The leaf fractions were ground in a knife mill (MA048, Marconi Equip., São Paulo, Brazil). Oven-dried samples were calcined using a Parr 6100 adiabatic calorimetric bomb (Parr Instrument Company, Moline, IL, USA). The calorimetric power of leaves (kcal) was estimated by calculating the sum of the respective mass fraction (ΔH°mass, kcal g^−1^) and area (ΔH°area, kcal m^−2^).

### 4.4. Infrared Gas Exchange and Chlorophyll A Fluorescence Analyses Parameters

An infrared gas exchange analyzer (IRGA) was used to measure 10- to 28-day-old experimental plants using the healthy, young, expanded leaf of the 4th or 5th leaf (counting downwards from the apical meristem). An IRGA (LI-6800, Li-Cor Inc., Lincoln, NE, USA) coupled with a Multiphase Flash^TM^ Fluorometer (LI-6800-01; Li-Cor Inc., Lincoln, NE, USA) was used according to the manufacturer’s light source: red:blue ratio (90:10), 2000 µmol m^−2^ s^−1^ light source, 400 µmol mol^−1^ constant in the chamber sample (CO_2__sample), 60% sample chamber relative humidity (%RH_sample), flow rate of 700 µmol s^−1^, fan speed of 10,000 rpm, and 25 °C heat exchanger temperature of the sample (Theat_sample) with a 6 cm^2^ sample chamber coupled to chlorophyll *a* fluorescence measurements simultaneously for all parameters.

Chlorophyll *a* fluorescence was measured in leaves previously acclimated (12 h) to the dark to measure “dark acclimated” fluorescence parameters (initial fluorescence, Fo, and maximum fluorescence, Fm). Variable fluorescence (Fv) was estimated using the equation Fv = Fm − Fo, allowing an estimation of the Fv/Fm ratio (maximum quantum efficiency of PSII photochemistry). Other chlorophyll *a* fluorescence measurements were performed on light acclimated leaves using the multiphase flash fluorescence protocol (MPF) with a saturating intensity of 15,000 µmol m^−2^s^−1^, dark modulation rate of 5 kHz, and light modulation rate of 50 kHz. Maximum Chl fluorescence (Fm’) was measured at 250 kHz during the saturating pulse, and fluorescence was detected at >700 nm (Li-Cor Inc.). The PSII maximum efficiency (Fv’/Fm’), quantum yield of photosystem II photochemistry (Φ PSII), electron transport rate through photosystem PSII (ETR) (µmol m^−2^ s^−1^), non-photochemical quenching (NPQ), photochemical quenching factor (qP) and non-photochemical quenching factor (qN) were estimated using LiCor^®^ software simultaneously with gas exchange measurements of light [68,69].

### 4.5. OJIP Chlorophyll a Fluorescence Transient

Chlorophyll *a* fluorescence transient induction was performed on the same leaves as “classical” fluorescence measurements using an LI-6800 instrument. Before being measured, each selected leaf was acclimated in the dark overnight (12 h). Afterward, a clipping chamber and a saturating light pulse of 15,000 µmol m^−2^ s^−1^ were applied for 1 s in induction mode, which closed all of the reaction centers, and the following fluorescence parameters were measured [31,70]: SFI, Structure function index; PI_(abs)_, Performance index for energy conservation from photons absorbed by PSII antenna until the reduction of PSI acceptors; D.F., Driving force on light absorption by a leaf cross-section. Data analysis and equations for calculating JIP-test parameters are explained in a previous study [69,71]. Biolyzer software version 4.0^®^ (Laboratory of Bioenergetics. University of Geneva; Geneva, Switzerland) was used to estimate the JIP-test parameters associated with the electron transport chain in plants.

### 4.6. Hyperspectral Optical Leaf Properties

Tobacco adaxial leaf spectral reflectance data were obtained using a FieldSpec 3 Jr. spectroradiometer (ASD Inc., Boulder, CO, USA) with a spectral resolution of 3 nm between 350 and 1400 nm and 10 nm between 1400 and 2500 nm. The equipment was programmed to perform 50 readings for each sample, thereby generating an average reflectance hyperspectral curve. Readings (scans) were recorded with the plant-probe device connected to the spectroradiometer by an optical fiber to avoid atmospheric influences and external interference. The plant probe has an artificial light consisting of a 4.5 W halogen lamp, which allows its operation regardless of the light conditions in the laboratory, greenhouse and environmental measures. The plant-probe device has an internal 99% white reference (i.e., Spectralon^®^; ASD Inc., Boulder, CO, USA) that is used as a reflectance standard, and an opaque background of 1% reflectance was used in the plant probe to ensure the collection of pure reflectance spectra from the leaf-based methods following [2,58].

### 4.7. Statistical Analyses

#### 4.7.1. Descriptive Analysis

All statistical analyses of yield, calorimetric energy and chlorophyll *a* fluorescence were performed using Excel 2021^®^ (Microsoft Office Inc., Sunnyvale, CA, USA), Statistica^®^ 12.0 software (Statsoft Inc., Tulsa, OK, USA), The Unscramber X10.4^®^ (Camo Software, Oslo, Norway) and the R package R-Core-Team (2020) (https://www.R-project.org; accessed on 15 August 2022) [55].

#### 4.7.2. Statistical Analyses of the Leaf Spectral Signature

The Shapiro-Wilk test was used to assess the normal distribution of data, and Bartlett’s test was used to assess the homogeneity of variance of hyperspectral indices, yield, calorimetric energy and chlorophyll *a* fluorescence parameters derived from the hyperspectral data using the PLSR models. Data transformation was not needed. The effects of light qualities (white, blue, red, and red-plus-far-red) on the leaf traits were analyzed using one-way analysis of variance (ANOVA). Duncan’s post-hoc test was used. The effects of light on the untransformed reflectance profiles (averaged per plant) were assessed using PERMANOVA by employing Euclidian measurements of dissimilarity using the Euclidean distance with the “vegan” package in R-Core Team (2020).

#### 4.7.3. Principal Component Analysis (PCA)

Principal component analysis (*p* < 0.05) was performed using “The Unscrambler X” software, version 10.4 (CAMO AS, Oslo, Norway) as an indicator of whether the variance in the reflectance hyperspectral between the varieties could be explained or not and how effectively tobacco plant varieties could be grouped. The numbers of components based on the highest average accuracy (Acc) and *Kappa* (K) values obtained for the validation models from partial least squares regression (PLSR) are reported. Partial least squares discriminant analysis was performed with the “caret” and “vegan” packages in R-Core Team (2020).

#### 4.7.4. Partial Least Squares Regression (PLSR) Analysis of Reflectance Data

The data were subjected to a Shapiro–Wilk test to assess the normal distribution and homogeneity of variance with Bartlett’s test and to obtain the prediction models of quality (yield, calorimetry energy and chlorophyll *a* fluorescence). No transformation was needed. For all variables, the marginal wavelengths recorded (350–399 and 2401–2500 nm), which are the nosiest ones, were removed to improve the accuracy of the data. Afterward, the data (tobacco hyperspectral reflectance) were centered on the mean and subjected to a partial least squares regression (PLSR) analysis. The algorithm for model inputs was NIPALS, and output outlier limits were defined by Leverage’s type and analyzed using Leverage and Hotelling’s T^2^ test (limit of 5%). For that purpose, the spectral data of the 260 samples of different parameters collected were divided into two groups. The first group consisted of 70% (182) of the samples with the aim of creating the model (training), while the second group was represented by 30% (78) of the remaining samples with the aim of testing (prediction) the adjusted PLSR model. These samples were randomly selected from the dataset, avoiding bias that might influence the quality of the model following [4].

The calibration (Cal) and leave-one-out cross-validation (Cva) methods were used to predict the quality attributes based on the yield, calorimetry energy and chlorophyll *a* fluorescence (i.e., yield, biomass of tobacco plants (g plant^−1^); ΔH°mass (kcal g^−1^), calorimeter energy content by mass; ΔH°area (kcal m^−2^), calorimetry energy content by area; Fv/Fm: Maximum quantum efficiency of PSII photochemistry in the dark-acclimated state; Fv’/Fm’: Maximum efficiency of PSII in light-acclimated state; ETR, electron transport rate (µmol m^−2^ s^−1^); NPQ, non-photochemical quenching calculated as (Fm − Fm’)/Fm’; qP: Photochemical quenching calculated as (Fm’–Fs)/(Fm’ − Fo’); qN: Non-photochemical quenching calculated as (Fm − Fm’)/(Fm − Fo’); ΦPSII, PSII operating efficiency under light conditions as calculated as (1 − Fs/Fm’); P, fraction of light absorbed in PSII antennae that is utilized in PSII photochemistry; D, fraction of light absorbed in PSII antennae that is dissipated thermally; SFI, Structure function index; PI_(abs)_, Performance index for energy conservation from photons absorbed by PSII antenna until the reduction of PSI acceptors; D.F., Driving force on light absorption by the leaf cross-section).

The predictive ability of the calibration models was evaluated by calculating metrics such as R^2^ (coefficient of determination), offset, RMSE (root mean square error) and RPD (ratio of performance to deviation), and bias was determined to assess the quality and accuracy of the model. According to [66,72], R^2^ values are classified as R^2^ < 0.50 (models with poor predictions incapable of distinguishing high and low values), R^2^ between 0.50 and 0.65 (models with moderate predictions that indicate the possibility of discriminating high and low concentrations in the model), R^2^ between 0.66 and 0.81 (good prediction models that facilitate quantitative predictions), R^2^ between 0.82 and 0.90 (very good quantitative prediction models) and R^2^ ≥ 0.91 (excellent prediction models). In addition, [72] suggest three categories for the RPD [RPD = 1/√(1 − R^2)]: (A) excellent models (RPD > 2.0); (B) useful models (1.4 < RPD < 2.0); and (C) unreliable models (RPD < 1.4) [5,11,41]. β-Coefficients, Y = β0 + β1λ1 + … + βnλn + ε, for parameters obtained with hyperspectral data for reflectance (400 at 2400 nm) are displayed following the method proposed in [4].

These randomized investigations generated a distribution of fit statistics simplifying the evaluation of model stability, as well as uncertainty in the model predictions. The strength contribution of PLSR loadings by individual wavelengths was also assessed using the VIP selection statistics, which highlight the importance of individual wavelengths in explaining the variation in both the response and predictor variables: larger weights provide higher values to the contribution of individual wavelengths to the predictive model [7]. The modeling approach and the data analyses were performed using the “pls” package in R-Core Team (2020) following [7].

In addition, before developing the prediction model, all preliminary models were investigated to identify poorly predicted outliers. However, prediction residuals were explored to identify potential outliers. Following the method proposed following [7], the spectral profiles of outliers were further examined for errors (e.g., elevated reflectance in the Vis wavelengths, spectral jumps produced by misaligned detector splicing, concave spectral shape at the red-edge peak), all likely due to the operational errors during spectral measurements (in reference or target collections) samples [7]. The standard measurements of the outliers were also examined for extremes in the data distribution. The outliers removed accounted for approximately 10% of the initial data, consistent with previous studies [4,7].

We also performed external validation by applying PLSR coefficients to a dataset independent from the one used for calibration and validation (30% of the whole dataset). Relations between predicted and observed values were tested by performing a regression analysis, and fit statistics (i.e., R^2^, Offset, RMSE, SEP, RPD, and bias) were investigated to assess the accuracy of the model-derived estimate (Figure 5 and Figure 6).

## 5. Conclusions

As shown in the present study, tobacco grown under light with distinct qualities has unique spectral signatures, as well as typical inflections of the -C-H and -N-H stretching vibrations from aromatic rings associated with the main compounds present in tobacco. In addition, some ChlF parameters, such as Fv/Fm, Fv’/Fm’, ETR, NPQ, qP, ΦPSII, P and D, were efficiently estimated using these Vis-NIR-SWIR spectra.

We were able to adjust PLSR models in the prediction (test) phase with R^2^_P_ and RPD_P_ values >0.82 and >3.33, respectively, for many variables predicted based on the full spectrum (400–2400 nm) with hyperspectral techniques. The most important wavelengths for the construction of the PLSR model of the evaluated parameters were well distributed within the three operating ranges of the spectroradiometer (Vis-NIR-SWIR). According to the regression coefficients, the most important bands were generally close to 400 (violet), 440 (blue), 550 (green), 670 (red), 700–750 (red edge), 1330 (NIR), 1450 (SWIR), 1940 (SWIR) and 2200 (SWIR) nm.

This study confirms the potential of a Vis-NIR-SWIR spectroradiometer to estimate the physiological parameters (yield, ΔH°area, ΔH°mass, Fv/Fm, Fv’/Fm’, ETR, NPQ, qP, qN, ΦPSII, P, D, SFI, PI_(abs)_ and D.F.) in tobacco plants. Therefore, this technique is a promising alternative for the routine analysis of the aforementioned parameters, as it provides advantages such as rapid data acquisition over a wide range. Based on the information provided above, since the Vis-NIR-SWIR spectroradiometer facilitates data acquisition and processing, it can be directly applied by field or plant physiologists (fast inline classification) with an excellent capacity for monitoring of the photodevelopment and understanding photosystem dynamics in tobacco, and other, plants.

## Figures and Tables

**Table 1:** Descriptive analysis performed in tobacco plants. Parameters: Yield, biomass of tobacco plants (g plant^−1^); ΔH°mass (kcal g^−1^), calorimeter energy content by mass; ΔH°area (kcal m^−2^), calorimeter leaf energy content by area; Fv/Fm: Maximum quantum efficiency of PSII photochemistry in the dark-acclimated state; Fv’/Fm’: Maximum efficiency of PSII in light-acclimated state; ETR, electron transport rate (µmol m^−2^ s^−1^); NPQ, non-photochemical quenching calculated as (Fm − Fm’)/Fm’; qP: Photochemical quenching calculated as (Fm’–Fs)/(Fm’ − Fo’); qN: Non-photochemical quenching calculated as (Fm − Fm’)/(Fm − Fo’); ΦPSII, PSII operating efficiency in light conditions as calculated (1 − Fs/Fm’); P, fraction of light absorbed in PSII antennae that is utilized in PSII photochemistry; D, fraction of light absorbed in PSII antennae that is dissipated thermally; SFI, Structure function index; PI_(abs)_, Performance index For energy conservation photons absorbed by PSII antenna, until the reduction of PSI acceptors; D.F., Driving force on absorption light by cross-section leaf.

Parameter	Count (n)	Mean	Median	Minimum	Maximum	CV (%)
Yield (g plant^−1^)	260	4.98	4.86	1.12	9.13	48.77
ΔH° (kcal g^−1^)	260	11.27	8.03	1.80	24.88	58.98
ΔH° (kcal m^−2^)	260	0.36	0.27	0.03	0.84	65.04
Fv/Fm	260	0.86	0.86	0.76	0.95	6.00
Fv’/Fm’	260	0.66	0.66	0.59	0.71	3.70
ETR	260	104.54	99.29	69.23	145.21	19.43
NPQ	260	0.50	0.49	0.20	0.85	30.17
qP	260	0.74	0.74	0.60	0.87	9.90
qN	260	0.40	0.40	0.23	0.55	15.77
ΦPSII	260	0.51	0.50	0.39	0.60	11.53
P	260	0.49	0.48	0.37	0.59	12.03
D	260	0.34	0.34	0.29	0.41	7.03
SFI	260	1.24	1.28	0.79	1.69	21.87
PI_(abs)_	260	14.00	12.80	5.89	24.16	35.79
D.F.	260	2.57	2.55	1.77	3.18	14.34

**Table 2:** Statistical metrics obtained by the PLSR model in calibration and cross-validation. Maximum number of components used (factors PLS), model goodness-of-fit (R^2^), offset, root mean square error (RMSE) and ratio of performance to deviation (RPD) for calibration (Cal) and cross-validation (Cva) data generated using 260 random permutations of the data with 70% used for Cal and 30% used for Val for the PLSR models predicting parameters of yield, calorimetry and chlorophyll *a* fluorescence parameters from hyperspectral of tobacco leaves. Bias outputs are not shown, as they were always lower than 0.01 for both Cal and Cva. Parameter abbreviations—see Table 1. The bold represents statistically significant regression models (R^2^). The underline indicates a bad residual prediction deviation (RPD) was calculated.

Parameter	Maximum Factors PLS	Calibration				Cross-Validation			
		R^2^	Offset	RMSE	RPD	R^2^	Offset	RMSE	RPD
Yield (g plant^−1^)	5	**0.85**	0.75	0.93	2.61	0.84	0.82	1.04	2.5
ΔH° (kcal g^−1^)	11	**0.91**	0.98	1.97	3.37	0.91	1.11	2.20	3.3
ΔH° (kcal m^−2^)	10	**0.93**	0.03	0.06	3.81	0.92	0.03	0.08	3.5
Fv/Fm	7	**0.82**	0.16	0.02	2.36	0.76	0.21	0.03	2.0
Fv’/Fm’	8	**0.84**	0.12	0.01	2.50	0.70	0.20	0.01	1.8
ETR	8	**0.91**	10.55	6.33	3.33	0.86	14.47	8.06	2.7
NPQ	2	0.62	0.18	0.08	1.63	0.61	0.19	0.09	1.6
qP	7	**0.93**	0.07	0.02	3.78	0.91	0.09	0.03	3.3
qN	3	0.29	0.29	0.05	1.19	0.22	0.32	0.05	1.1
ΦPSII	8	**0.91**	0.04	0.02	3.39	0.91	0.06	0.02	3.3
P	8	**0.94**	0.03	0.01	4.12	0.91	0.04	0.02	3.4
D	6	**0.72**	0.10	0.01	1.89	0.64	0.12	0.01	1.7
SFI	6	**0.70**	0.38	0.15	1.81	0.64	0.45	0.17	1.7
PI_(abs)_	6	**0.61**	5.72	3.27	1.59	0.54	6.61	3.72	1.5
D.F.	5	**0.65**	0.92	0.21	1.68	0.57	1.12	0.25	1.5

**Table 3:** Statistical metrics from the PLSR model in the predicted phase. Maximum number of components used (factors PLS), model goodness-of-fit (R^2^), offset, standard error of prediction (SEP), ratio of performance to deviation (RPD) and linear equation prediction (Ŷ) to base models (a prediction using an independent sample coupling to calibrated models) parameters of yield, calorimetry and chlorophyll *a* fluorescence parameters from hyperspectral data of date tobacco leaves. Bias outputs are not shown, as they were always lower than 0.01 to predict regression analysis. Parameter abbreviations—see Table 1. The bold represents statistically significant regression models (R^2^). The underline indicates a bad residual prediction deviation (RPD) was calculated.

Parameter	Maximum Factors PLS	Predicted				
		R^2^	Offset	SEP	RPD	Linear Equation Prediction to Calibration Model (R^2^_P_)
Yield (g plant^−1^)	5	**0.71**	0.80	1.33	1.86	Ŷ = 0.8875x + 0.5701
ΔH° (kcal g^−1^)	11	**0.85**	0.95	2.66	2.58	Ŷ = 0.8224x + 0.8697
ΔH° (kcal m^−2^)	10	**0.87**	0.03	0.08	2.77	Ŷ = 0.9344x + 0.0194
Fv/Fm	7	**0.81**	0.17	0.03	2.29	Ŷ = 0.9922x + 0.0075
Fv’/Fm’	8	**0.56**	0.07	0.01	1.51	Ŷ = 0.6338x + 0.2408
ETR	8	**0.91**	11.38	6.74	3.33	Ŷ = 0.9915x + 0.6146
NPQ	2	**0.67**	0.12	0.07	1.74	Ŷ = 0.8813x + 0.0631
qP	7	**0.93**	0.07	0.02	3.78	Ŷ = 1.0043x − 0.0021
qN	3	0.32	0.22	0.04	1.21	Ŷ = 0.7419x + 0.1093
ΦPSII	8	**0.92**	0.04	0.02	3.54	Ŷ = 0.9657x + 0.0202
P	8	**0.94**	0.03	0.01	4.08	Ŷ = 1.0065x − 0.0308
D	6	**0.62**	0.11	0.01	1.62	Ŷ = 0.9154x + 0.0308
SFI	6	0.53	0.45	0.19	1.46	Ŷ = 0.8253x + 0.2124
PI_(abs)_	6	0.44	6.14	3.93	1.34	Ŷ = 0.7410x + 3.3400
D.F.	5	0.45	0.86	0.29	1.35	Ŷ = 0.6797x + 0.8462

**Table 4:** The most responsive variable importance for projection (VIP) by wavelengths selected according to the regression coefficient for quality parameter for the PLSR model prediction. Parameter abbreviations—see Table 1.

Parameter	Selection	Most Responsive VIP by Wavelength (nm)
Yield (g plant^−1^)	8	440, 550, 672, 702, 935, 1404, 1590, 1922
ΔH° (kcal g^−1^)	17	401, 454, 518, 548, 682, 702, 742, 865, 998, 1114, 1331, 1387, 1430, 1530, 1712, 1927, 2048
ΔH° (kcal m^−2^)	12	401, 544, 663, 684, 703, 743, 998, 1391, 1532, 1679, 1885, 2031
Fv/Fm	11	405, 482, 544, 680, 705, 752, 1143, 1406, 1594, 1894, 2197
Fv’/Fm’	9	405, 484, 550, 676, 702, 730, 1419, 1440, 2192
ETR	9	400, 483, 534, 664, 698, 724, 1332, 1440, 1873
NPQ	5	402, 545, 712, 770, 1287
qP	7	440, 527, 668, 702, 1343, 1395, 1873
qN	5	550, 713, 1095, 1297, 1433
ΦPSII	6	405, 481, 663, 702, 723, 1327
P	8	403, 478, 668, 697, 730, 1332, 1874, 1920
D	7	405, 515, 550, 630, 698, 752, 2156
SFI	11	405, 445, 524, 552, 675, 701, 730, 1446, 1610, 1929, 2200
PI_(abs)_	9	405, 435, 515, 550, 668, 700, 730, 1586, 1930
D.F.	10	400, 405, 440, 521, 674, 701, 730, 1332, 1569, 1927

## Data Availability

All data included in the main text.

## Abbreviations List

Acc: Average accuracy; ANOVA: Analysis of variance; B: Blue light; ChlF: Chlorophyll *a* fluorescence; D.F.: Driving force on light absorption by cross-section leaf; D: Fraction of light absorbed in PSII antennae that is dissipated thermally; ETR: Electron transport rate (µmol m^−2^ s^−1^); Fo: Minimum fluorescence yield of the dark-adapted state; Fo’: Minimum fluorescence intensity of the light-adapted state; Fm: Maximum fluorescence yield of the dark-adapted state; Fm’: Maximum fluorescence intensity of the light-adapted state; FR: Far-red light (red added to far-red light); Fs: Steady state fluorescence intensity in light-adapted state; Fv/Fm: Maximum quantum efficiency of PSII photochemistry in the dark-acclimated state; Fv’/Fm’: Maximum efficiency of PSII in light-acclimated state; K: *Kappa* coefficient; NIR: near-infrared spectral region; NPQ: Non-photochemical quenching calculated as (Fm − Fm’)/Fm’; P: Fraction of light absorbed in PSII antennae that is utilized in PSII photochemistry; PAM: Pulse-amplitude modulation; PC: Principal component; PCA: Principal component analysis; PERMANOVA: Permutational analysis of variance; PI_(abs)_: Performance index for energy conservation from photons absorbed by the PSII antenna until the reduction of PSI acceptors; PLSR: Partial least squares regression; PSI: Photosystem I; PSII: Photosystem II; qN: Non-photochemical quenching calculated as (Fm − Fm’)/(Fm − Fo’); qP: Photochemical quenching calculated as (Fm’-Fs)/(Fm’ − Fo’); R: Red light; R^2^: Coefficient of determination; RMSE: Root mean square error; RPD (ratio of performance to deviation); SFI: Structure function index; STEPWISE: Stepwise regression is a method of fitting regression models in which the choice of predictive variables is carried out by an automatic procedure; SWIR: Shortwave infrared spectral region; VIP: Variable importance to the projection; Vis: Visible spectral region; W: While light; ΔH°: Calorimetric energy; ΦPSII: PSII operating efficiency in light conditions as calculated (1 − Fs/Fm’).
